# Beyond cure: patient reported outcomes of hepatitis C treatment among people who inject drugs in Australia

**DOI:** 10.1186/s12954-018-0248-4

**Published:** 2018-08-15

**Authors:** Annie Madden, Max Hopwood, Joanne Neale, Carla Treloar

**Affiliations:** 1 Centre for Social Research in Health, UNSW, Sydney, Australia; 2 National Addiction Centre, King’s College London, London, UK

**Keywords:** Patient reported, Consumer involvement, Outcomes, Experience, People who inject drugs, Hepatitis C

## Abstract

**Background:**

Recent advances in the treatment of hepatitis C virus (HCV) infection provide the possibility of eliminating HCV as a public health threat. This focus on HCV elimination through treatment, however, is also driving a concomitant focus on ‘achieving cure’ as the primary outcome of treatment. The aim of this paper is to explore what people who inject drugs consider to be important in relation to outcomes of HCV treatment, and whether there are outcomes ‘beyond cure’ that might be important to understand as part of improving engagement in treatment.

**Methods:**

A peer researcher with experience of both HCV treatment and injecting drug use conducted interviews with 24 people in the following groups in Melbourne, Australia: (1) people who had refused or deferred HCV treatment; (2) people who were actively thinking about, planning and/or about to commence HCV treatment; (3) people currently undertaking HCV treatment and (4) people who had recently completed HCV treatment.

**Results:**

The findings show that people who inject drugs are seeking outcomes ‘beyond cure’ including improved physical and mental health, positive changes in identity and social relationships and managing future health and risk. Participants indicated that these other outcomes had not been addressed within their experience of HCV treatment.

**Conclusion:**

While cure is an obvious outcome of HCV treatment, patients are seeking change in other areas of their lives. This study also provides valuable insights for the development of patient-reported measures in this context, which would be an important step towards more patient-centred approaches to HCV treatment.

## Background

In recent years, there have been significant advances in therapeutic efficacy and tolerability of hepatitis C virus (HCV) treatment medications [1]. Direct acting antiviral (DAA) therapies are more efficacious and of shorter duration, with a lower side effect profile, than previous longstanding interferon-based treatments [2, 3]. These advances open up the possibility of HCV elimination as a public health challenge [4]. The main risk factor for HCV infection in Australia and many other countries is the use of unsterile equipment for drug injection [5], making people who inject drugs a key group to engage in HCV treatment.

The possibility of HCV elimination (as a public health challenge) drives a focus on cure (i.e. sustained virological response) as the key outcome of treatment. The focus on curative HCV treatments also draws from the ‘cascade of care’ concept which seeks to describe and quantify each step in the spectrum of engagement in care and treatment [6]. In HCV literature, the constructions of care cascades overwhelmingly conclude with a measure of the proportion of treated individuals who achieve a cure [7, 8]. What gets measured and reported in these cascades are a pragmatic mix of available data (such as from routine surveillance or from records of prescriptions) and often supported by mathematical modelling (which again draws upon available data). People who inject drugs and who are living with HCV have not had the opportunity to critique and inform this cascade to include measures important to them, such as outcomes other than cure.

While attaining a cure is undoubtedly the main rationale behind people having HCV treatments, affected people may prioritise other outcomes. For example, there is an emergent literature exploring what people who inject drugs expect and hope from DAA therapies, beyond cure. One longitudinal qualitative study conducted in the UK asked people undergoing a mix of interferon-based and DAA therapies for their views on treatment benefit [9]. Participants identified a range of social benefits, including social reconnection, social redemption and a return to ‘normality’. This study also found important differences between discourses and debates that emphasise the public health benefits of HCV treatment and the benefits as perceived by people undertaking treatment.

Differences between patient and provider views of treatment outcomes have been widely documented across the health sector [10]. To identify and better understand these differences, there has been increasing international interest in the development and use of patient reported measures (PRMs). PRMs focus on what matters to patients rather than to clinicians or other stakeholders. They allow patients to evaluate the success of health interventions and provide feedback to healthcare providers about the issues that matter to them as patients [11–15].

Despite the growing use of PRMs in many areas of medicine [16], they have received remarkably little attention within the alcohol and other drug (AOD) sector. This likely reflects the fact that, historically, people who use drug treatment services have not tended to be consulted in treatment decision making [17] and not seen as ‘credible’ patients. Moreover, injecting drug use can produce a master identity status, which colours perception of all other aspects of these patients’ lives [18]. In this context, stigma and discrimination can also influence whether or not people who inject drugs will seek DAA therapies and, among those who do, what their experiences of treatment are [19].

The aim of this paper is to explore what people who inject drugs consider to be the important outcomes of HCV treatment beyond cure in order to better understand their engagement with DAA therapies, and also to identify factors that might be relevant to the development of a new PRM for HCV treatment.

## Methods

This study adopted a participatory approach. A participatory approach seeks to understand experience and knowledge through a process of collective inquiry, collaboration and reflection [20, 21]. The participatory design of this study included the engagement of a peer researcher (AM, first author) with direct experience of both injecting drug use and HCV treatment with DAAs. The involvement of a peer researcher was also useful for creating a ‘safe space’ for the exploration of the subjective experiences of people who inject drugs (a highly marginalised community) in an era of rapidly expanding biomedical responses to HCV infection.

Semi-structured interviews were conducted with 24 people who self-reported living with HCV, were 18 years and over, and who had a recent history of injecting drug use. Participants were recruited through community-based networks of people who inject drugs in Melbourne, Australia, in collaboration with a community partner organisation, Harm Reduction Victoria. Six participants were recruited across each of four groups: (1) people who had refused or deferred treatment for HCV with DAAs; (2) people who were actively thinking about, planning and/or about to commence treatment for HCV with DAAs; (3) people currently undertaking treatment for HCV with DAAs and (4) people who had recently completed treatment for HCV with DAAs.

Interviews were conducted between December 2016 and March 2017, were of 20–30 min duration, and were all conducted by the peer researcher. Participants were asked to provide a biographical snapshot including their experience of diagnosis, knowledge and expectations of HCV treatment, current health and well-being, future/life following treatment and basic demographic information. Each participant was paid AUD $20 for their time and expertise.

The interviews were audio-recorded, transcribed verbatim and edited to remove any identifying information. The first phase of data analysis was conducted using an interpretative thematic analysis approach based on an iterative, inductive process whereby three members of the research team read each transcript to identify recurring themes and patterns of meaning within the data [22]. These team members then met to discuss and agree upon the most prominent themes and patterns which were then each assigned a code. From this process, a coding framework was developed based on the key thematic categories, and the transcripts were re-read by the peer researcher to identify any further instances of the key themes within the data. The third phase of analysis involved identifying relevant narrative excerpts from each interview transcript which were then added into the coding document under the relevant thematic category. This document was then used as the basis for the development of this paper.

## Results

Participants included nine women and 15 men with an age range of 28–64 years. Two participants identified as Aboriginal or Torres Strait Islanders. Three participants reported unstable housing and eight participants were living in public housing. Most (*n* = 15) received government benefits as their main source of income, and eight participants reported full-time or part-time employment. Nine participants reported completing high school education to year 12 or above (see Table 1 for further details). Themes pertaining to DAA treatment outcomes beyond cure comprised physical and psychological health, understanding clinical markers and future liver health, issues regarding reinfection, identity and social connections and stigma and discrimination.

**Table 1:** Stage 1—demographic characteristics of participants by group (total *n* = 6 participants per group)

	Group 1	Group 2	Group 3	Group 4	Total (*n* = 24)
Women	3	3	2	1	9
Men	3	3	4	5	15
Age range (years)	32–64	28–54	32–58	33–63	28–63
Aboriginal and/or Torres Strait Islander	1	1	0	0	2
Unstable housing	1	1	1	0	3
Public housing	1	3	2	2	8
Government benefits as main source of income	3	4^a^	4	4^a^	15
Regular employment (f/t or p/t)	3	2	2	1	8
Completed high school education^b^	1	3	2	3	9

^a^One person receiving government support for full-time study

^b^Completed year 12 or above in the Australian education system

People who had refused or deferred treatment for HCV with DAAs (i.e. group 1 participants, *n* = 6) were asked during interviews to comment on the reasons for declining DAA treatment, rather than reporting their expectations and hopes ‘beyond cure’. As such, data from group 1 were not included in the analysis for this paper but instead are presented in a forthcoming publication about the barriers to DAA treatment [Beyond interferon side effects: what residual barriers exist to DAA hepatitis C treatment for people who inject drugs? forthcoming]. In summary, data from group 1 highlighted a variety of participants’ personal and structural vulnerabilities that were significant barriers to DAA treatment, including difficulties in acquiring a HCV diagnosis, poor venous access to provide sufficient blood for DAA testing, poor mental health, ongoing problems related to drug dependence, unstable housing and past experiences of healthcare-related stigma and discrimination.

To meet the aims of this paper, the findings below are drawn from the three groups of participants who either were preparing for DAA treatment, having treatment or had completed treatment.

### Better physical and mental health after HCV DAA treatment and hopes for a healthier future

Some participants had expectations of how HCV treatment would make them feel physically, psychologically and emotionally in the immediate post-treatment period such as feeling better, sleeping better, eating better, feeling more positive and being healthier. Participants who had either recently completed treatment themselves or knew others who had recently completed treatment, spoke about the transformative effect of clearing the virus on people’s health, energy levels and even on their overall appearance: ‘almost like they were sparkling’.

Since treatment, I have a lot more energy, I’m much more active, I feel a lot happier. (male, group 4, 33 years)

One participant, who had previously undertaken unsuccessful interferon-based HCV treatments, described the effect of finally clearing the virus following treatment with DAAs as ‘amazing’ and ‘like lifting a veil type thing’. Others explained that the outcome they valued most was reduced concern about the impact of chronic HCV infection on their liver health into the future.

It’s not so much about becoming healthier, it’s about trying to prevent… it’s more of a preventative measure for me than becoming healthier. I’m hoping that by doing treatment and taking the sort of tax off my liver, that will enhance my health in the future, as opposed to making me healthier now. (female, group 3, 58 years)

For some participants, however, the ‘promises’ and expectations associated with clearing the virus they had lived with for decades had not transpired. Some were disappointed that a sustained virological response had not lived up to their expectations of feeling better, of having more energy and being able to get ‘on with life’ and ‘make up for lost time’. Indeed, some participants stated that although they initially felt great after completing treatment, this feeling did not last and they now found themselves feeling quite depressed and ‘let down’ both by the ‘hype’ and broader expectations of a ‘better life’ promised from a cure.

### Understanding liver health and infectiousness and having a plan for post-treatment care

Participants with moderate to serious liver damage, including cirrhosis, reported that they did not have sufficient information about the ongoing risk of liver disease following viral clearance. Participants seemed to be largely unaware, and uninformed, of the potential for ongoing liver disease (including a possible life-long risk of liver cancer), and a need for ongoing monitoring of liver health for those diagnosed with moderate to serious fibrosis and/or cirrhosis prior to commencing treatment. Of the participants who were currently undergoing DAA treatment or who had recently completed DAA treatment, none were aware of any plans for ongoing liver health management including the need for ongoing liver function tests or liver ultrasounds. This is highlighted by the response of one participant who, despite stating he had diagnosed liver damage of at least medium level fibrosis prior to commencing treatment with the DAAs and was at least 6 months post treatment at the time of interview, responded in the following way when asked about whether anyone on his treatment team had spoken with him about the need for ongoing monitoring:

No, they haven’t asked. Nobody’s really… yeah I don’t think at the time there’s any call for monitoring beyond SVR12. (male, group 4, 56 years)

Related to this issue was the lack of information, and clarity, about whether participants would remain ‘infectious’ following viral clearance. The lack of an explanation regarding HCV infectiousness, ‘beyond cure’, was a problem for some people in this study who wanted to be certain they could no longer transmit the virus to others, including their children and grandchildren, because following successful treatment for HCV, people will remain HCV antibody positive despite being HCV PCR negative. Several participants spoke about the relief and how ‘nice’ it was not to have to worry about their blood in the health care setting due to the risk of stigma, but also in the household context and not having to be concerned about potentially exposing family and friends to hepatitis C. Some of these same participants, however, also spoke about a lingering doubt in relation to infectiousness post treatment. For example, in response to being asked about whether she would feel the need to tell people about having had hepatitis C in the past now that she had cleared the virus, one participant said:

I reckon I need to clarify that because, I think you still *have* got a low level, haven’t you, of something in your system? (female, group 4, 63 years; participant emphasis added)

### Creating a new identity and enhancing social connections

The potential positive impact of curing HCV for a person’s identity and their sense of self when in social interactions with friends, family and in society generally was raised by most participants. Participants explained that being cured of HCV removed the stress of disclosing HCV status. Participants expressed residual shame or internalised stigma about having an infection, or being treated for an infection, that is widely associated with injecting drug use. Participants described the relief of not having to manage their daily interactions and routines in order to avoid the stigma associated with the infection. For example, in anticipation of life after DAA therapy, some simply welcomed the sense of relief that ‘being hep C free’ would bring, particularly with close friends and family, whom they believed would view them more favourably. Others felt that being ‘hep C free’ would mean that they could also leave the ‘drug user’ label behind—not because they necessarily had stopped using drugs, but because clearing HCV infection would, among many things, present an opportunity to create a new, virally untainted identity.

It would be nice not to always have to say, “I have hep C” or “I’m a hep C carrier” or however you put it and it doesn’t really need to be something that I need to disclose, because I still have that feeling of “oh so, to have got hep C even though there are other ways of getting hep C, it always brings me back to being that drug user”. It’s not something that I’m ashamed of, but it’s not something that I want as part of my life, you know running with me parallel for the whole of my life. (female, group 3, 58 years)

I won’t feel like I’m diseased. Like I’m carrying around something that is dirty to the world… You know what I mean? I won’t be scared about meeting people anymore and talking to them because I have nothing to hide. At the moment I am hiding two things and living a lie and I don’t want to do that anymore. Once I’m hep C free I can move on. (female, group 2, 41 years)

Some participants anticipated that the outcomes of HCV treatment (such as enhanced emotional health and well-being and impact on identity) would have a positive impact on their intimate relationships and their ability to better meet family and work commitments.

I feel bad for my kids, because I want them to have a mum to be proud of. I want to be able to take them out, I want to be able to go to the school and talk to the other mums, but I feel ashamed of myself and I feel like I’m not up to the standard they deserve. (female, group 2, 41 years)

Participants identified a variety of interpretations of ‘hep C free’. For example, they described clearing HCV infection in terms of ‘moving on’ to a life of better health, a new identity and expanded opportunities for social connections. Conversely, clearing the infection was also viewed as losing something intrinsic to one’s identity, particularly given that HCV is a long-term, slow progressing chronic disease that most participants had lived with for numerous decades. People who had lived with HCV for many years reported a range of affective responses from clearing their infection:

I don’t think it’s something that clinicians and that think about. That for some people it is a massive thing and it’s kind of like mourning … it’s like when you stop using drugs and you kind of mourn that … It’s like a huge part of your identity so then to let that go, then you have to fill that with something else and you’ve got to find that energy somewhere else. …but I think that’s the same with any chronic illness. Surely that’s a thing, surely some shrink has put it in blog or something by now. (female, group 3, 42 years)

### Equipped to avoid or manage new infections

Most participants in this study held strong views on the issue of HCV risk after treatment. While some stated they would feel upset and angry with themselves if they were to become reinfected, others said they would be shocked and surprised: ‘I can’t imagine how that would happen’. Most participants said that they would not feel guilty if they acquired another HCV infection. A few made the distinction between feeling guilty and feeling embarrassed about acquiring HCV again, but most said that it would not prevent them from coming forward for retreatment because it ‘is what it is’ and re-treatment would be ‘much more important than any embarrassment I might feel about becoming reinfected’. Those who did express reluctance in coming forward should they acquire HCV again did so mostly out of concern for the high cost to the health system of the new treatments.

Participants were also highly confident that they could avoid reinfection. The belief that they were extremely unlikely to be reinfected stemmed largely from their ability to access information and services (including needle and syringe programs) in a way that was not the case when they first acquired the virus. For others, however, safer injecting was firmly embedded in their routine practice, such that even contemplating acquiring a new HCV infection seemed almost confrontational.

It’s not likely to happen. It’s *really* unlikely to happen! Um, in fact if it did happen, something would have seriously fucked up for me. Something *really* significant will have happened in my decision-making processes to allow that, that risk, to happen. Yeah that would be pretty significant. (male, group 2, 50 years; participant emphasis added)

Despite the high level of confidence among participants in relation to avoiding reinfection following successful HCV treatment, participants identified a general lack of discussion and information on harm reduction and safer injecting practices in HCV treatment services and associated AOD health services. For example, in response to being asked if healthcare professionals had discussed the potential for reinfection with him following successful HCV treatment, and what he might need to do to ensure he remained hepatitis C free, this participant responded by saying that no one had raised this issue with him:

I think ‘cause they know me so well. I’ve known them all for years – most of them. They already sort of know me and they know the way that I do things. They know that they didn’t need to bring that sort of thing up maybe… I dunno… maybe… but I think that would be a factor. (male, group 4, 39 years).

## Discussion

DAA therapies cure HCV infection in a majority of cases. This is an advance in clinical treatment that can support goals to drastically reduce HCV morbidity and mortality. Like any condition, cure is only one aspect of therapy for the patient. In this study, people who injected drugs and were living with HCV articulated a range of outcomes that they valued including enhanced physical and mental health, avoiding a need to disclose HCV infection, better social relationships, changes in identity, a positive orientation to the future and being equipped to manage future health and risk. While the majority of people undergoing HCV DAA treatment will achieve cure, these other outcomes will not necessarily be achieved unless services reorientate to a more patient-centred approach beyond cure. Further, efforts to promote treatment should engage with the varying outcomes being sought by this group.

The clinical literature has established a benefit of SVR to health related quality of life [23] and higher quality of life scores among people undertaking DAA treatment compared with people experiencing earlier generation interferon-containing treatments [24]. These results may feed the ‘promise’ and ‘hype’ reported by participants in this study. Care should be taken when extrapolating research results to the experience of individual patients that the ‘promise’ of greater feelings of well-being do not become entrenched in clinical (or health promotion) scripts when describing treatment effects but that variability in response is acknowledged. Further, while DAAs are undoubtedly a very significant step forward in tolerability of treatment, a focus on comparing old (interferon-based) with new (interferon-free) might also feed the ‘hype’. For those who have not undertaken previous interferon-based treatments, these comparisons are irrelevant.

In this study, removing a need to disclose HCV infection was a valued outcome from DAA treatments. Disclosure of HCV infection is a potentially stressful event for affected people because the condition is closely associated with injecting drug use. Our findings indicated that among participants who had cleared HCV following DAA treatment, some were able to forge a new identity, and enhance their social connections [9], in part because a cure removed the stress of having to disclose HCV. Indeed, the stress of disclosing HCV has been highlighted in previous research where negative reactions have been documented following disclosure, especially in healthcare settings [25]. This is a key concern given that the promotion of DAA treatments is central to elimination efforts aimed at curbing future HCV-related morbidity, mortality and healthcare costs associated with liver disease. The fear of HCV-related stigma and discrimination from within the healthcare sector following HCV disclosure decreases the likelihood that affected people will access healthcare settings to commence DAA treatments. Our findings regarding disclosure corroborate those of other studies and an Australian state-government enquiry, over the past 20 years [26–28].

Given the stigma associated with HCV (and by associated, drug use), it may be surprising that some participants expressed concern that they would experience a loss of identity in being ‘hep C free’. To identify as a person with HCV who injects drugs is a political act, due to the socio-legal sanctions ascribed to the social practice of injecting drug use [29] and the perceived threat to community represented by infectious disease [30]. Historically, people with identities and practices that are perceived as threatening, and which are subsequently marginalised by institutions such as healthcare, have at times effectively organised political action and extended support to fellow ‘travellers’, creating movements and moments where a sense of solidarity through common purpose and shared hope for the future prevail [31–33]. Throughout both the HIV and HCV epidemics, for example, substantial strides were made by highly organised, international groups of substance users in relation to harm reduction, drug law reform, the visibility of drug users, their rights to respect and the right to healthcare, among other social justice reforms [34]. It is not entirely surprising then that in some ways, for some people, HCV infection comes to represent a struggle for visibility, legitimacy and equality in a hostile world, and that being cured of the infection removes positive aspects of ‘otherness’ that affected people highly value, and when gone, profoundly miss.

While representations of the cascade of care typically conclude with ‘cure’ [7, 8], efforts to achieve elimination require ongoing efforts to prevent reinfection [35]. Participants in this study expressed strong concerns about the prospect of reinfection and were confident that they could avoid this. International studies show that the prevalence of reinfection among people who inject drugs is low, at less than 5 per 100 person years [36, 37]. Nevertheless, those who do acquire new infections tend to be those involved in injecting networks, since injecting places them at risk of acquisition and, possibly, transmission of hepatitis C. The cascade of care needs to be reformulated, or expanded, to address reinfection and ongoing liver damage if we are to capitalise on the opportunities that HCV DAA therapies provide. All patients should be offered support during and after treatment to avoid reinfection particularly in settings such as AOD treatment programs where patients may be expected or assumed to be abstinent, and there can be risks to admissions of ongoing drug use [38]. Additionally, there can be an ongoing risk of liver disease among people who achieve a HCV cure [39]. Preparing people during HCV treatment with information and a liver health plan can minimise the incidence and resultant costs of future liver disease. Rates of reinfection and liver disease post treatment are not currently included in HCV cascades of care.

These data were collected in Australia in which there is a universal access policy for HCV DAA therapy. However, experiences of prohibition and criminalization of injecting drug use are global, and issues of stigma and mistrust related to hepatitis C permeate the international literature [40]. Hence, the findings related to the themes of identity, perceived infectiousness and social connections may be applicable to other settings. Other settings which have specific limitations for treatment eligibility, access to prevention services and treatment for reinfection [41–43] may generate other outcomes for this patient group.

## Conclusion

The possibility of DAA treatments leading to elimination of HCV has excited the interests of many. However, there could be an inherent tension between the goals of HCV elimination projects and the outcomes that people who inject drugs value and expect from HCV treatment, which could undermine elimination efforts. Despite these very transactional concerns, it is no less important to understand what people who inject drugs want and need as outcomes of HCV treatment. This is an ethical proposition and the foundations of patient-centred responses to HCV that value community knowledge [44, 45]. The insights from this study could be used to underpin patient-reported measures for use in clinical and research settings to expand the ways in which HCV treatment is understood and valued.

## References

[1] Falade-Nwulia O, Suarez-Cuervo C, Nelson D, Fried M, Segal J, Sulkowski M (2017). Oral direct-acting agent therapy for hepatitis C virus infection: a systematic review. Ann Intern Med.

[2] Pawlotsky JM, Hepatitis C (2016). Drugs: is next generation the last generation?. Gastroenterology.

[3] Feld JJ, Foster GR (2016). Second generation direct-acting antivirals - do we expect major improvements?. J Hepatol.

[4] World Health Organisation (2016). Combating hepatitis B and C to reach elimination by 2030: advocacy brief.

[5] Hajarizadeh B, Grebely J, Dore G (2013). Epidemiology and natural history of hepatitis C virus infection. Nat Rev Gastroenterol Hepatol.

[6] Gardner E, McLees M, Steiner J, Del Rio C, Burman W (2011). The spectrum of engagement in HIV care and its relevance to test-and-treat strategies for prevention of HIV infection. Clin Infect Dis.

[7] Linas BP, Barter DM, Leff JA, Assoumou SA, Salomon JA, Weinstein MC (2014). The hepatitis C cascade of care: identifying priorities to improve clinical outcomes. PLoS One.

[8] Janjua NZ, Kuo M, Yu A, Alvarez M, Wong S, Cook D (2016). The population level cascade of care for hepatitis C in British Columbia, Canada:the BC hepatitis testers cohort (BC-HTC). EBioMedicine.

[9] Harris M (2017). Managing expense and expectation in a treatment revolution: problematizing prioritisation through an exploration of hepatitis C treatment 'benefit'. Int J Drug Policy.

[10] Trujols J, Portella MJ, Iraurgi I, Campins MJ, Siñol N (2013). Cobos JPdL. Patient-reported outcome measures: are they patient-generated, patient-centred or patient-valued?. J Ment Health.

[11] Greenhalgh J, Dalkin S, Gooding K, Gibbons E, Wright J, Meads D, et al. Health Services and Delivery Research. Functionality and feedback: a realist synthesis of the collation, interpretation and utilisation of patient-reported outcome measures data to improve patient care. Southampton (UK): NIHR Journals Library Copyright (c) Queen's Printer and Controller of HMSO 2017. This work was produced by Greenhalgh et al. under the terms of a commissioning contract issued by the secretary of state for health. This issue may be freely reproduced for the purposes of private research and study and extracts (or indeed, the full report) may be included in professional journals provided that suitable acknowledgement is made and the reproduction is not associated with any form of advertising. Applications for commercial reproduction should be addressed to: NIHR journals library, National Institute for Health Research, evaluation, trials and studies coordinating entre, alpha house, University of Southampton Science Park, Southampton SO16 7NS, UK.; 2017. 28121094

[12] Basch E (2017). Patient-reported outcomes - harnessing patients' voices to improve clinical care. N Engl J Med.

[13] Williams K, Sansoni J, Morris D, Grootemaat P, Thompson C (2016). Patient-reported outcome measures: literature review.

[14] Basch E (2014). New frontiers in patient-reported outcomes: adverse event reporting, comparative effectiveness, and quality assessment. Annu Rev Med.

[15] Black N. Patient reported outcome measures could help transform healthcare. BMJ : Br Med J. 2013;346 10.1136/bmj.f167. 10.1136/bmj.f167 23358487

[16] Dawson J, Neale J (2009). Measuring health status. Research methods for health and social care.

[17] Brener L, Resnick I, Ellard J, Treloar C, Bryant J (2009). Exploring the role of consumer participation in drug treatment. Drug Alcohol Depend.

[18] Lloyd C (2013). The stigmatization of problem drug users: a narrative literature review. Drugs: Educ, Prev Policy.

[19] Ahern J, Stuber J, Galea S (2007). Stigma, discrimination and the health of illicit drug users. Drug Alcohol Depend.

[20] Bergold J, Thomas S (2012). Participatory Research Methods: A Methodological Approach in Motion [110 paragraphs]. Forum Qualitative Sozialforschung/Forum: Qualitative Social Research.

[21] Wicks P, Reason P (2009). Initiating action research: challenges and paradoxes of opening communicative space. Action Res.

[22] Braun V, Clarke V (2006). Using thematic analysis in psychology. Qual Res Psychol.

[23] Smith-Palmer J, Cerri K, Valentine W (2015). Achieving sustained virologic response in hepatitis C: a systematic review of the clinical, economic and quality of life benefits. BMC Infect Dis.

[24] Younossi ZM, Stepanova M, Nader F, Lam B, Hunt S (2015). The patient's journey with chronic hepatitis C from interferon plus ribavirin to interferon- and ribavirin-free regimens: a study of health-related quality of life. Aliment Pharmacol Ther.

[25] Hopwood M, Nakamura T, Treloar C (2010). Disclosing hepatitis C infection within everyday contexts: implications for accessing support and healthcare. J Health Psychol.

[26] Anti-Discrimination Board of New South Wales (2001). C-Change: Report of the enquiry into hepatitis C related discrimination.

[27] Crofts N, Louie R, Loff B (1997). The next plague: stigmatization and discrimination related to hepatitis C virus infection in Australia. Health Hum Rights.

[28] Fry M, Bates G (2012). The tasks of self-managing hepatitis C: the significance of disclosure. Psychol Health.

[29] Room R (2005). Stigma, social inequality and alcohol and drug use. Drug Alcohol Rev.

[30] Douglas M (1994). Purity and danger: an analysis of the concepts of pollution and taboo.

[31] Roy CM, Cain R (2001). The involvement of people living with HIV/AIDS in community-based organizations: contributions and constraints. AIDS Care.

[32] Friedman SR, Des Jarlais DC, Sotheran JL, Garber J, Cohen H, Smith D (1987). AIDS and self-organization among intravenous drug users. Int J Addict.

[33] Wieloch N (2002). Collective mobilization and identity from the underground: the deployment of “oppositional capital” in the harm reduction movement. Sociol Q.

[34] Madden A, Wodak A (2014). Australia’s response to HIV among people who inject drugs. AIDS Educ Prev.

[35] Dowdle W (1998). The principles of disease elimination and eradication. Bull World Health Organ.

[36] Midgard H, Bjoro B, Maeland A, Konopski Z, Kileng H, Damas JK (2016). Hepatitis C reinfection after sustained virological response. J Hepatol.

[37] Weir A, McLeod A, Innes H, Valerio H, Aspinall EJ, Goldberg DJ (2016). Hepatitis C reinfection following treatment induced viral clearance among people who have injected drugs. Drug Alcohol Depend.

[38] Treloar C, Rance J, Dore G, Grebely J (2014). Barriers and facilitators of hepatitis C care and treatment uptake in opioid treatment programs: the ETHOS study in NSW, Australia. J Viral Hepat.

[39] Conti F, Buonfiglioli F, Scuteri A, Crespi C, Bolondi L, Caraceni P (2016). Early occurrence and recurrence of hepatocellular carcinoma in HCV-related cirrhosis treated with direct-acting antivirals. J Hepatol.

[40] Treloar C, Rhodes T (2009). The lived experience of hepatitis C and its treatment among injecting drug users: qualitative synthesis. Qual Health Res.

[41] Barua S, Greenwald R, Grebely J, Dore GJ, Swan T, Taylor LE (2015). Restrictions for medicaid reimbursement of Sofosbuvir for the treatment of hepatitis C virus infection in the United States. Ann Intern Med.

[42] Marshall A, Saeed S, Barrett L, Cooper C, Treloar C, Bruneau J (2016). Restrictions for reimbursement of direct-acting antiviral treatment for hepatitis C virus infection in Canada: a descriptive study. CMAJ Open.

[43] Marshall AD, Cunningham EB, Nielsen S, Aghemo A, Alho H, Backmund M (2018). Restrictions for reimbursement of interferon-free direct-acting antiviral drugs for HCV infection in Europe. Lancet Gastroenterol Hepatol.

[44] Hepworth J, Krug G (1997). Hepatitis C and policy implementation: ethics as a dialogic process for resource allocation [editorial]. Aust N Z J Public Health.

[45] Rose D (2014). Patient and public involvement in health research: ethical imperative and/or radical challenge?. J Health Psychol.

